# Effect of Small Dense Low‐Density Lipoprotein Cholesterol Combined With High‐Sensitivity C‐Reactive Protein on Cardiometabolic Multimorbidity: A National Cohort Study

**DOI:** 10.1002/clc.70296

**Published:** 2026-04-20

**Authors:** Zi Guo, Jiaying Shen, Feng Song

**Affiliations:** ^1^ Department of General Practice Huzhou Central Hospital, Fifth School of Clinical Medicine of Zhejiang Chinese Medical University Huzhou Zhejiang P.R. China; ^2^ Department of Geriatric Medicine Huzhou Central Hospital, Fifth School of Clinical Medicine of Zhejiang Chinese Medical University Huzhou Zhejiang P.R. China

**Keywords:** cardiometabolic multimorbidity, high‐sensitivity C‐reactive protein, risk, small dense low‐density lipoprotein cholesterol

## Abstract

**Background:**

Small dense low‐density lipoprotein cholesterol (sdLDL‐C) is a more significant atherosclerotic factor than LDL‐C. This investigation aimed to examine the correlation between sdLDL‐C levels and the risk of cardiometabolic multimorbidity (CMM) in middle‐aged and older adults.

**Methods:**

Participants aged ≥ 45 years who did not have CMM at baseline from the China Health and Retirement Longitudinal Study (CHARLS) data set were included in this cohort study. The baseline data of the participants were collected in 2011 and followed up until 2020. sdLDL‐C is calculated using a validated formula. The relationship of sdLDL‐C with CMM risk was examined using Cox regression analyses. The combined effect of high‐sensitivity C‐reactive protein (hsCRP) and sdLDL‐C on CMM risk was explored.

**Results:**

A total of 9616 participants were included, of whom 1814 (18.86%) had CMM and 7802 (81.14%) did not have CMM. Elevated sdLDL‐C levels were correlated with a higher risk of CMM (HR [95% CI] = 1.10 [1.04−1.15]). The interaction terms for sdLDL‐C and hsCRP presented that participants in the low sdLDL‐C and high hsCRP group (HR [95% CI] = 1.21 [1.03‐1.41]), the high sdLDL‐C and low hsCRP group (HR [95% CI] = 1.21 [1.08−1.36]), and the high sdLDL‐C and high hsCRP group (HR [95% CI] = 1.36 [1.15−1.62]) had a higher risk of CMM versus the low sdLDL‐C and low hsCRP group, and the trend for these interaction terms was significant (P_trend_ = 0.031).

**Conclusion:**

Elevated sdLDL‐C levels were related to an increased risk of CMM, and there was an interaction between sdLDL‐C and hsCRP on the increased risk of CMM.

## Graphical abstract

Elevated small dense low‐density lipoprotein cholesterol (sdLDL‐C) levels were associated with a higher risk of cardiometabolic multimorbidity (CMM). There was an interaction between sdLDL‐C and high‐sensitivity C‐reactive protein (hsCRP) on the increased risk of CMM.

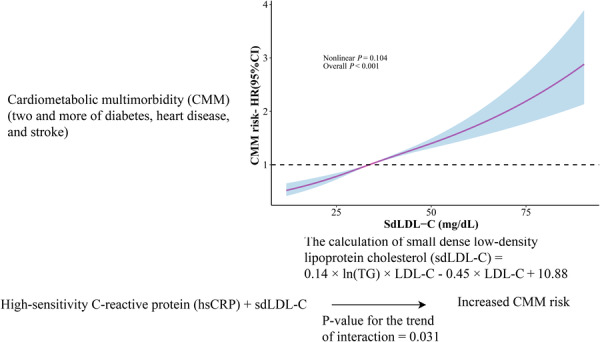

## 1 Introduction

Cardiometabolic diseases (CMD) represent a leading contributor to the global disease burden and mortality, and the prevalence of CMD is gradually increasing with the aging of the population [1]. Defined as the simultaneous presence of two or more of diabetes, heart disease, and stroke, cardiometabolic multimorbidity (CMM) constitutes a highly common pattern of comorbidity [2, 3]. An additional CMD has been reported in 22% of diabetic patients, and the prevalence of CMM is higher in patients with heart disease and stroke [4]. The presence of CMM is connected to a significantly higher risk of mortality and a substantial decrease in life expectancy relative to a single CMD [5]. Therefore, exploring the factors related to the development of CMM is of great clinical value in preventing CMM and reducing the burden of disease.

Low‐density lipoprotein cholesterol (LDL‐C) is strongly related to the development and progression of cardiovascular disease (CVD). Elevated LDL‐C levels are correlated with an increased risk of CVD [6, 7] and diabetes [8]. However, studies have shown that even when LDL‐C levels are controlled within reasonable limits, cardiovascular risk remains in participants [9]. This suggests a need to explore markers that are more strongly associated with CMD risk. Small dense LDL‐C (sdLDL‐C) is a subtype of LDL‐C (with reduced particle size and increased density). sdLDL‐C exhibits higher atherosclerotic properties due to its prolonged circulation time, enhanced endothelial permeability, and oxidative susceptibility as compared to LDL‐C [10, 11]. Numerous investigations have demonstrated that sdLDL‐C concentrations exhibit a stronger correlation with the risk of coronary heart disease in comparison to LDL‐C levels [12, 13]. Furthermore, sdLDL‐C is a better predictor of stroke and coronary heart disease development than LDL‐C [14, 15]. However, the relationship between sdLDL‐C and CMM risk has not been reported. In addition, the inflammatory state of the body, characterized by high‐sensitivity C‐reactive protein (hsCRP), is also an important influencing factor of CMD [16, 17]. Several studies have demonstrated that the coexistence of high hsCRP levels with dyslipidemia leads to a higher risk of CVD and stroke [18, 19]. There may be a combined impact between hsCRP and sdLDL‐C on CMM. Thus, this investigation sought to explore the connection between sdLDL‐C levels and the risk of CMM among middle‐aged and older adults based on nationally representative longitudinal data, and to investigate the combined effect of hsCRP and sdLDL‐C on CMM risk.

## 2 Methods

### 2.1 Study Design and Populations

This cohort study adopted data from the China Health and Retirement Longitudinal Study (CHARLS) datasets between 2011 and 2020. CHARLS is a national longitudinal survey employing a multistage stratified sampling design to assess the social, economic, and health profiles among adults aged 45 years and above in China (https://charls.charlsdata.com/index/zh-cn.html). The CHARLS study recruited a total of 17,708 middle‐aged and older adult participants from 28 provinces, 150 regions/districts, and 450 villages throughout China. Baseline data collection was initiated in 2011, followed by four follow‐up surveys administered in 2013, 2015, 2018, and 2020. The specific research protocols and data collection processes employed in CHARLS have been thoroughly documented in earlier published investigations [20]. Ethical approval for the CHARLS study was granted by the Ethics Committee of Peking University, with all participants providing signed informed consent. Participants recorded in CHARLS 2011 were included in this study. The exclusion criteria of participants were: (1) those aged ≤ 45 years; (2) those with missing triglycerides (TG) data; (3) those with missing LDL‐C data; (4) those with CMM at baseline; and (5) those with missing follow‐up data.

### 2.2 Outcome

The outcome of this study was the occurrence of CMM during follow‐up, which was defined as participants who simultaneously suffered from two or more of heart disease, stroke, and diabetes by the time of follow‐up in 2020 [21, 22]. The identification of heart disease and stroke was based on self‐report of previous clinical diagnoses. The recognition of diabetes was based on self‐report and laboratory tests (fasting blood glucose ≥ 7.0 mmol/L or glycated hemoglobin [HbA1c] ≥ 6.5%).

### 2.3 Measurement of sdLDL‐C and hsCRP

sdLDL‐C levels are calculated using a published formula [23]: sdLDL‐C = 0.14*ln(TG)*LDL‐C−0.45*LDL‐C + 10.88. When sdLDL‐C was analyzed in terms of categorical variables, the CMM‐based Survcutpoint() function was applied to filter the best cutoff value for classification (< 37.669, ≥ 37.669 mg/dL). hsCRP levels were obtained directly from laboratory test data at baseline. hsCRP was classified into < 3 mg/L and ≥3 mg/L based on the cut‐off values reported in previous studies [24].

### 2.4 Data Collection

Baseline data were collected from participants covering age, gender, education, marital status, residence, annual income, insurance, body mass index (BMI), hsCRP, TG, LDL‐C, depression, hypertension, dyslipidemia, kidney disease, arthritis, smoking, drinking, physical activity, sleep duration, social activity, grip, health status, and estimate glomerular filtration rate (eGFR). Smoking was categorized as non‐heavy smoking and heavy smoking, with heavy smoking defined as ≥ 20 cigarettes per day [25]. Drinking was classified as less than twice a day and more than twice a day. BMI was categorized as <24 kg/m^2^ and ≥24 kg/m^2^ (overweight or obesity). The identification of hypertension, dyslipidemia, and kidney disease was based on self‐report and laboratory tests (hypertension [systolic blood pressure ≥ 130 mmHg or diastolic blood pressure ≥ 6.5 mmHg]; dyslipidemia [total cholesterol ≥ 200 mg/dL (5.2 mmol/L), TG ≥ 150 mg/dL (1.7 mmol/L), LDL‐C ≥ 130 mg/dL (3.4 mmol/L), HDL‐C ≤ 40 mg/dL (1.0 mmol/L)]; kidney disease [eGFR < 60 mL/min/1.73 m^2^]).

### 2.5 Statistical Analysis

For quantitative data, the distribution was described using mean ± standard deviation (SD) or median [Q1, Q3], and group comparisons were performed with the *t*‐test, *t*'‐test, or Wilcoxon rank‐sum test. Categorical data were summarized as frequencies and percentages (*n* [%]), and the Chi‐square test or Fisher's exact test was adopted for analysis. Covariates with <20% missing data were imputed utilizing multiple imputation, while those exceeding 20% missing data were excluded. The difference analysis before and after interpolation of missing values was conducted (Supporting Information S1: Table 1).

Confounders correlated with CMM were selected using weighted univariable Cox regression, and variables with *p* < 0.05 in univariable analyses were screened for final confounders after bidirectional stepwise regression with the Akaike Information Criterion (AIC) minimization rule (Supporting Information S1: Table 2). The relationships of sdLDL‐C with CMM were examined using univariable and multivariable Cox regression analyses, and results were presented as hazard ratio with 95% confidence interval (HR [95% CI]). Multivariable Cox analysis adjusted for residence, BMI, hsCRP, depression, hypertension, dyslipidemia, arthritis, health status, eGFR, and CMD at baseline. Further analyses were performed according to age, gender, BMI, hypertension, dyslipidemia, kidney disease, and baseline CMD subgroups. A restricted cubic spline (RCS) curve was applied to examine the non‐linear relationships between sdLDL‐C and CMM. The Kaplan−Meier (KM) curves were utilized to investigate the correlation between sdLDL‐C and CMM. Furthermore, the combined effect of sdLDL‐C and hsCRP on CMM risk was also explored. Statistical analyses were achieved using R 4.4.3 software (Institute for Statistics and Mathematics, Vienna, Austria), considering *p*‐value < 0.05 as significant.

## 3 Results

### 3.1 Baseline Characteristics

**Figure 1:**
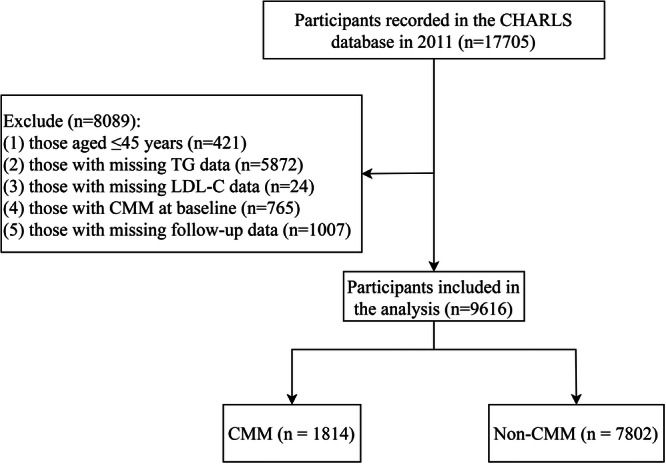
Flowchart of the screening process for the study population. CHARLS, the China Health and Retirement Longitudinal Study database; CMM, cardiometabolic multimorbidity; LDL‐C, low‐density lipoprotein cholesterol; TG, triglycerides.

The CHARLS database recorded baseline data of 17 705 participants in 2011. Following the exclusion of 8089 individuals, the analysis comprised 9616 eligible participants (Figure 1). The baseline characteristics of these 9616 participants are presented in Table 1. The mean age of participants was 58.59 (±8.96) years, and females accounted for 5146 (53.51%). The mean sdLDL‐C level was 35.20 (±11.55) mg/dL, and 6073 (63.16%) participants had sdLDL‐C levels < 37.669 mg/dL. For hsCRP, 8029 (83.50) participants had hsCRP levels < 3 mg/L, and 1587 (16.50) participants had hsCRP levels ≥ 3 mg/L. At baseline, 6916 (71.92%) participants did not have CMD, 1224 (12.73%) had diabetes only, 846 (8.80%) had CVD only, and 630 (6.55%) had stroke only. By follow‐up completion, 1814 (18.86%) participants had CMM, and 7802 (81.14%) did not have CMM. The mean follow‐up time was 8.28 (±1.96) years. Furthermore, there were significant differences between CMM and non‐CMM patients in age, gender, residence, BMI, hsCRP, depression, hypertension, dyslipidemia, kidney disease, arthritis, smoking, drinking, physical activity, sleeping duration, grip, health status, eGFR, TG, LDL‐C, and CMD at baseline, and follow‐up time (*p* < 0.05).

**Table 1:** Baseline characteristics of participants.

Variables	Total (*N* = 9616)	Non‐CMM (*N* = 7802)	CMM (*N* = 1814)	*p*
Age, years, Mean (±SD)	58.59 (±8.96)	58.35 (±8.99)	59.62 (±8.74)	< 0.001
*Gender, n (%)*				< 0.001
Male	4470 (46.49)	3712 (47.58)	758 (41.79)	
Female	5146 (53.51)	4090 (52.42)	1056 (58.21)	
Education, *n* (%)				0.117
Primary school and below	6630 (68.95)	5371 (68.84)	1259 (69.40)	
Junior high school	1961 (20.39)	1617 (20.73)	344 (18.96)	
High school and above	1025 (10.66)	814 (10.43)	211 (11.63)	
*Marital status, n (%)*				0.099
Married or cohabiting	8598 (89.41)	6996 (89.67)	1602 (88.31)	
No married	1018 (10.59)	806 (10.33)	212 (11.69)	
*Residence, n (%)*				0.001
Village	8674 (90.20)	7076 (90.69)	1598 (88.09)	
Town/city	942 (9.80)	726 (9.31)	216 (11.91)	
*Annual income, n (%)*				0.475
< 10 000	1712 (17.80)	1398 (17.92)	314 (17.31)	
≥ 10 000	1660 (17.26)	1330 (17.05)	330 (18.19)	
Unknown	6244 (64.93)	5074 (65.03)	1170 (64.50)	
*Insurance, n (%)*				0.094
No	554 (5.76)	434 (5.56)	120 (6.62)	
Yes	9062 (94.24)	7368 (94.44)	1694 (93.38)	
*BMI, kg/m^2^, n (%)*				< 0.001
< 24	5643 (58.68)	4762 (61.04)	881 (48.57)	
≥ 24	3973 (41.32)	3040 (38.96)	933 (51.43)	
*hsCRP, mg/L, n (%)*				< 0.001
< 3	8029 (83.50)	6601 (84.61)	1428 (78.72)	
≥ 3	1587 (16.50)	1201 (15.39)	386 (21.28)	
*Depression, n (%)*				< 0.001
No	6081 (63.24)	5096 (65.32)	985 (54.30)	
Yes	3535 (36.76)	2706 (34.68)	829 (45.70)	
*Hypertension, n (%)*				< 0.001
No	4661 (48.47)	3984 (51.06)	677 (37.32)	
Yes	4955 (51.53)	3818 (48.94)	1137 (62.68)	
*Dyslipidemia, n (%)*				< 0.001
No	3404 (35.40)	2946 (37.76)	458 (25.25)	
Yes	6212 (64.60)	4856 (62.24)	1356 (74.75)	
*Kidney disease, n (%)*				< 0.001
No	8864 (92.18)	7232 (92.69)	1632 (89.97)	
Yes	752 (7.82)	570 (7.31)	182 (10.03)	
*Arthritis, n (%)*				< 0.001
No	8023 (83.43)	6582 (84.36)	1441 (79.44)	
Yes	1593 (16.57)	1220 (15.64)	373 (20.56)	
*Smoking, n (%)*				0.033
No heavy	7988 (83.07)	6450 (82.67)	1538 (84.79)	
Heavy	1628 (16.93)	1352 (17.33)	276 (15.21)	
*Drinking, n (%)*				0.029
Less than twice a day	9123 (94.87)	7383 (94.63)	1740 (95.92)	
More than twice a day	493 (5.13)	419 (5.37)	74 (4.08)	
*Physical activity, n (%)*				0.030
Insufficient	667 (6.94)	516 (6.61)	151 (8.32)	
Sufficient	3335 (34.68)	2726 (34.94)	609 (33.57)	
Unknown	5614 (58.38)	4560 (58.45)	1054 (58.10)	
*Sleeping duration, hours, n (%)*				0.020
6−9	4392 (45.67)	3610 (46.27)	782 (43.11)	
≤ 6	4833 (50.26)	3868 (49.58)	965 (53.20)	
> 9	391 (4.07)	324 (4.15)	67 (3.69)	
*Social activity, n (%)*				0.205
No	4754 (49.44)	3882 (49.76)	872 (48.07)	
Yes	4862 (50.56)	3920 (50.24)	942 (51.93)	
*Grip, n (%)*				0.001
High	8428 (87.65)	6882 (88.21)	1546 (85.23)	
Low	1188 (12.35)	920 (11.79)	268 (14.77)	
*Health status, n (%)*				< 0.001
Good	1720 (17.89)	1512 (19.38)	208 (11.47)	
Fair	4008 (41.68)	3279 (42.03)	729 (40.19)	
Poor	1805 (18.77)	1294 (16.59)	511 (28.17)	
Unknown	2083 (21.66)	1717 (22.01)	366 (20.18)	
eGFR, mL/min/1.73 m^2^, Mean (±SD)	96.40 (±13.68)	96.94 (±13.36)	94.10 (±14.72)	< 0.001
*CMD at baseline, n (%)*				< 0.001
No CMD	6916 (71.92)	6217 (79.68)	699 (38.53)	
Diabetes	1224 (12.73)	633 (8.11)	591 (32.58)	
Heart disease	846 (8.80)	466 (5.97)	380 (20.95)	
Stroke	630 (6.55)	486 (6.23)	144 (7.94)	
sdLDL‐C, mg/dL, Mean (±SD)	35.20 (±11.55)	34.52 (±11.23)	38.12 (±12.42)	< 0.001
*sdLDL‐C, mg/dL, n (%)*				< 0.001
< 37.669	6073 (63.16)	5126 (65.70)	947 (52.21)	
≥ 37.669	3543 (36.84)	2676 (34.30)	867 (47.79)	
TG, mg/dL, M (Q₁, Q₃)	105.32 (75.22, 153.99)	101.78 (73.46, 147.57)	124.79 (86.73, 182.31)	< 0.001
LDL‐C, mg/dL, Mean (±SD)	116.59 (±34.61)	115.95 (±33.96)	119.31 (±37.17)	< 0.001
Follow time, years, Mean (±SD)	8.28 (±1.96)	9.00 (±0.00)	5.19 (±2.93)	< 0.001

Abbreviations: BMI, body mass index; CMM, cardiometabolic multimorbidity; CMD, cardiometabolic diseases; eGFR, estimate glomerular filtration rate; hsCRP, high‐sensitivity C‐reactive protein; LDL‐C, low‐density lipoprotein cholesterol; sdLDL‐C, small dense low‐density lipoprotein cholesterol; TG, triglycerides.

### 3.2 The Relationship Between sdLDL‐C and CMM Risk

Table 2 lists the correlation of sdLDL‐C with CMM risk. High sdLDL‐C levels were related to an elevated CMM risk in both unadjusted (HR [95% CI] = 1.29 [1.24−1.34]) and adjusted (HR [95% CI] = 1.10 (1.04−1.15]) models. When sdLDL‐C was categorized by cutoff value (37.669 mg/dL), sdLDL‐C ≥ 37.669 mg/dL was related to a higher risk of CMM (adjusted HR [95% CI] = 1.19 [1.07_1.33]) versus sdLDL‐C < 37.669 mg/dL. After further adjusting lipid‐lowering drugs based on the adjusted model, sdLDL‐C ≥ 37.669 mg/dL remained associated with elevated CMM risk (HR [95% CI] = 1.19 [1.07−1.33]). For the predictive value of sdLDL‐C on CMM risk, sdLDL‐C (C‐index: 0.581 [95% CI: 0.567−0.595]) exhibited better predictive ability for CMM risk compared to LDL‐C (C‐index: 0.525 [95%CI: 0.511−0.540]) (*p* < 0.001).

**Table 2:** The relationship of sdLDL‐C with CMM risk.

Variables	Model 1		Model 2		Model 3	
	HR (95% CI)	*p*	HR (95% CI)	*p*	HR (95% CI)	*p*
sdLDL‐C	1.29 (1.24−1.34)	< 0.001	1.10 (1.04−1.15)	< 0.001	1.10 (1.04−1.15)	< 0.001
sdLDL‐C						
< 37.669 mg/dL	Ref		Ref		Ref	
≥ 37.669 mg/dL	1.65 (1.51−1.81)	< 0.001	1.19 (1.07−1.33)	0.001	1.19 (1.07−1.33)	0.001

*Note:* Model 1 is a univariable Cox regression analysis; Model 2 is a multivariable Cox regression analysis adjusted for residence, BMI, hsCRP, depression, hypertension, dyslipidemia, arthritis, health status, eGFR, and CMD at baseline; Model 3 is a multivariable Cox regression analysis further adjusted for lipid‐lowering drugs based on model 2.

Abbreviations: CI, confidence interval; CMM, cardiometabolic multimorbidity; HR, hazard ratio; Ref, reference; sdLDL‐C, small dense low‐density lipoprotein cholesterol.

**Figure 2:**
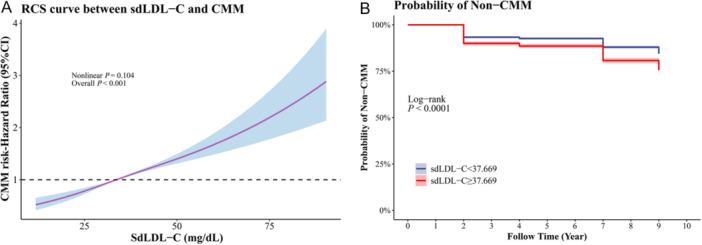
The relationship between sdLDL‐C levels and CMM risk. (A) The restricted cubic spline (RCS) curve between sdLDL‐C and CMM; (B) the Kaplan−Meier (KM) curve between sdLDL‐C and CMM. CMM, cardiometabolic multimorbidity; sdLDL‐C, small dense low‐density lipoprotein cholesterol.

Figure 2 presents the non‐linear relationship (Figure 2A) and KM curves (Figure 2B) between sdLDL‐C and CMM. The RCS curve exhibited that the non‐linear relationship between sdLDL‐C and CMM risk was not significant (P_overall_ < 0.001, P_non‐linear_ = 0.104), but elevated sdLDL‐C levels were associated with higher CMM risk. The KM curves demonstrated that individuals with sdLDL‐C ≥ 37.669 mg/dL were more likely to have CMM versus those with sdLDL‐C < 37.669 mg/dL (log‐rank *p* < 0.0001).

**Figure 3:**
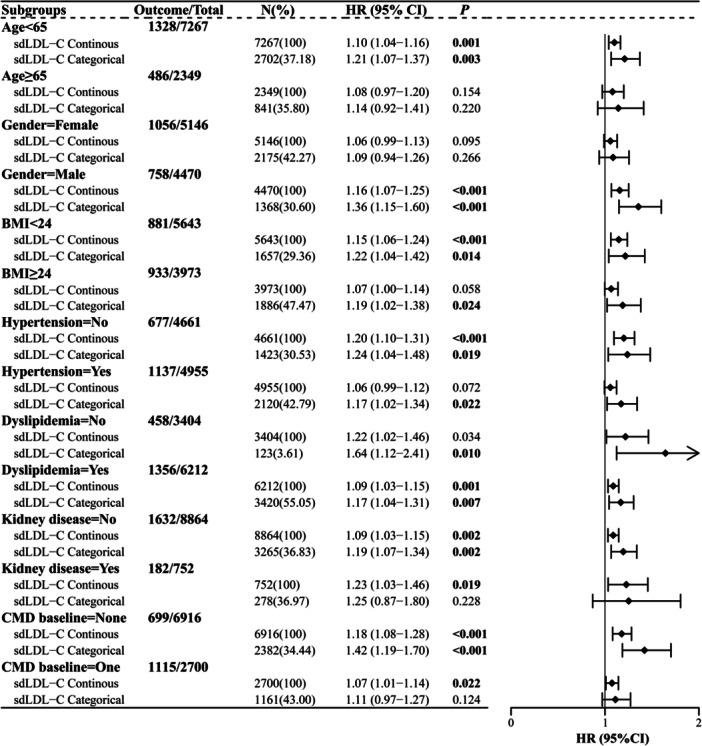
The associations between sdLDL‐C and CMM risk in different subgroups. CMD, cardiometabolic diseases; CMM, cardiometabolic multimorbidity; sdLDL‐C, small dense low‐density lipoprotein cholesterol. sdLDL‐C categorical, ≥ 37.669 versus < 37.669 mg/dL.

The associations of sdLDL‐C with CMM risk in different subgroups are displayed in Figure 3. Individuals with sdLDL‐C ≥ 37.669 mg/dL (vs. < 37.669 mg/dL) were related to an increased risk of CMM in those aged < 65 years (HR [95% CI] = 1.21 [1.07−1.37]), males (HR [95% CI] = 1.36 [1.15−1.60]), those with BMI < 24 kg/m^2^ (HR [95% CI] = 1.22 [1.04−1.42]) or BMI ≥ 24 kg/m^2^ (HR [95% CI] = 1.19 [1.02−1.38]), those with hypertension (HR [95% CI] = 1.17 [1.02−1.34]) or non‐hypertension (HR [95% CI] = 1.24 [1.04−1.48]), those with (HR [95% CI](HR [95% CI] = 1.17 [1.04−1.31]) or without (HR [95% CI] = 1.64 [1.12−2.41]) dyslipidemia, those without kidney disease (HR [95% CI] = 1.19 [1.07−1.34]), and those without CMD at baseline (HR [95% CI] = 1.42 [1.19−1.70]), but not in those aged ≥ 65 years (*p* = 0.220), females (*p* = 0.266), those with kidney disease (*p* = 0.228), and those with one CMD at baseline (*p* = 0.124).

### 3.3 The Combined Effect of sdLDL‐C and hsCRP on CMM Risk

Table 3 presents the combined effect of sdLDL‐C and hsCRP on CMM risk. Individuals with hsCRP ≥ 3 mg/L (HR [95% CI] = 1.17 [1.04−1.31]) were related to a higher risk of CMM versus hsCRP < 3 mg/L. The interaction terms for sdLDL‐C (low: <37.669, high: ≥37.669 mg/dL) and hsCRP (low: <3, high: ≥3 mg/L) demonstrated that individuals in the low sdLDL‐C and high hsCRP group (HR [95% CI] = 1.21 [1.03−1.41]), the high sdLDL‐C and low hsCRP group (HR [95% CI] = 1.21 [1.08−1.36]), and the high sdLDL‐C and high hsCRP group (HR [95% CI] = 1.36 [1.15−1.62]) had a higher risk of CMM versus the low sdLDL‐C and low hsCRP group, and the trend for these interaction terms was significant (P_trend_ = 0.031).

**Table 3:** The combined effect of sdLDL‐C and hsCRP on CMM risk.

Variables	Univariable analysis		Multivariable analysis	
	HR (95% CI)	*p*	HR (95% CI)	*p*
*sdLDL‐C*				
< 37.669 mg/dL (low)	Ref		Ref	
≥ 37.669 mg/dL (high)	1.65 (1.51−1.81)	< 0.001	1.19 (1.07−1.33)	0.002
*hsCRP*				
< 3 mg/L (low)	Ref		Ref	
≥ 3 mg/L (high)	1.43 (1.28−1.60)	< 0.001	1.16 (1.04−1.30)	0.010
Interaction				
Low sdLDL‐C & Low hsCRP	Ref		Ref	
Low sdLDL‐C & High hsCRP	1.44 (1.23−1.69)	< 0.001	1.21 (1.03−1.41)	0.021
High sdLDL‐C & Low hsCRP	1.66 (1.49−1.84)	< 0.001	1.21 (1.08−1.36)	0.002
High sdLDL‐C & High hsCRP	2.27 (1.93−2.65)	< 0.001	1.36 (1.15−1.62)	< 0.001
*P* for trend	0.031			

*Note:* Multivariable Cox regression analysis adjusted for residence, BMI, hsCRP, depression, hypertension, dyslipidemia, arthritis, health status, eGFR, and CMD at baseline.

Abbreviations: CI, confidence interval; CMM, cardiometabolic multimorbidity; HR, hazard ratio; hsCRP, high‐sensitivity C‐reactive protein; sdLDL‐C, small dense low‐density lipoprotein cholesterol.

## 4 Discussion

CMM causes a much higher disease burden than a single CMD. This investigation explored the correlation between sdLDL‐C levels and CMM risk. The findings revealed that elevated sdLDL‐C levels were associated with a higher risk of CMM. Further analyses indicated that the association between sdLDL‐C levels and CMM risk was also observed in different subgroups. Furthermore, there was an interaction between sdLDL‐C and hsCRP on the increased risk of CMM.

Co‐morbidities not only impose a severe disease burden but may also lead to increased or accelerated severity of one or more of the diseases, or the development of new diseases. CMM has been found to be correlated to cognitive function decline [2], dementia [26], as well as an increased risk of mortality [5]. Some factors related to the risk of CMM have been identified, such as lipids [27], abdominal obesity [28], insulin resistance [29], and a healthy lifestyle [30]. Among these CMM‐related lipids, elevated levels of TG and LDL‐C were correlated with an increased risk of CMM [27]. Nonetheless, sdLDL‐C appears to be a stronger prognostic marker for both coronary heart disease and stroke than conventional LDL‐C [14, 15]. The current investigation examined the correlation between sdLDL‐C levels and CMM risk. The results revealed that elevated sdLDL‐C levels were correlated with a higher CMM risk. Furthermore, hsCRP represents a common inflammatory biomarker that has been linked to a higher risk of CMM among middle‐aged and elderly populations [21]. Our results also confirmed that elevated hsCRP was connected with a higher risk of CMM. The interaction between sdLDL‐C and hsCRP on the increased risk of CMM was also observed in our findings. Previous studies have shown an interaction between dyslipidemia and hsCRP on the increased risk of CVD [18], which further confirms our results.

The exact mechanisms of elevated sdLDL‐C levels on increased CMM risk remain unclear. Several studies have reported that the effect of lipids on CMM risk may be correlated with obesity, lipid metabolism, inflammation, insulin resistance, and cardiovascular damage [28, 31]. The levels of sdLDL‐C have been shown to be highly correlated with metabolic disorders, obesity and inflammation [32, 33]. Obesity may increase CMM risk through endothelial dysfunction, atherosclerosis, inflammation, and insulin resistance [28]. Hormones (e.g., lipocalin and leptin) as well as cytokines (TNF‐α and IL‐1β) produced by adipocytes induce the development of insulin resistance and promote the progression of CMD [34]. Disturbances in lipid metabolism due to insulin resistance may cause imbalances in the protein kinase B (PKB) and mitogen‐activated protein kinase (MAPK) pathways, and metabolic byproducts may also affect insulin signaling, contributing to myocardial injury and stiffness [35, 36]. Insulin resistance leads to apoptosis of vascular smooth muscle cells, promoting atherosclerosis and advanced plaque formation [37]. Furthermore, insulin resistance stimulates the production of pro‐inflammatory factors and elicits oxidative stress, which disrupts myocardial architecture and metabolism, thus contributing to diastolic dysfunction and the development of heart failure [36, 38]. Various proteins (e.g., PCSK9, NRP1, and CD27) play roles in lipid metabolism and vascular regulation, thereby impacting vascular homeostasis and angiogenesis and driving the progression of CMD to CMM [39]. The small size of sdLDL‐C facilitates its penetration into the subendothelial space and renders it more susceptible to qualitative modifications such as oxidation, glycation, and glycosylation [40]. These modifications trigger inflammatory responses, enhance sdLDL‐C affinity for endothelial proteoglycans, promote preferential uptake by macrophages, accelerate foam cell formation, and contribute to atherosclerotic plaque development [41, 42]. Additionally, inflammation levels are significantly related to atherosclerosis, and inflammation plays an important role in influencing lipid metabolism and increasing insulin resistance [43, 44]. This might also be one of the explanations for the interaction between sdLDL‐C and hsCRP on the risk of CMM.

This investigation evaluated the association between sdLDL‐C and CMM risk utilizing data from a nationally representative cohort, providing additional evidence for the relationship between lipids and CMM. Nevertheless, some limitations need to be noted. First, the participants in the survey were individuals aged 45 or above, which limited the generalizability of the findings to those younger than 45 years of age. Second, although we have adjusted for confounders, there are still some confounders not measured by the database (e.g., diet) that may have influenced the results. Third, we calculated sdLDL‐C levels using formulas due to the complexity of laboratory measurements of sdLDL‐C levels. Although these formulas have been proven to accurately reflect the level of sdLDL‐C, there are still differences from the actual level of sdLDL‐C. Fourth, the diagnosis of heart disease and stroke mainly relies on self‐reporting, which may introduce recall biases and misclassification risks in the diagnosis of these conditions.

## 5 Conclusions

High sdLDL‐C levels were related to an increased risk of CMM. Moreover, there was an interaction between sdLDL‐C and hsCRP on the increased risk of CMM. Future studies may need to explore the relationship between more lipid components and CMM risk.

## Author Contributions

Z.G. and J.S. designed the study. Z.G. wrote the manuscript. F.S. collected, analyzed, and interpreted the data. J.S. critically reviewed, edited, and approved the manuscript. All authors read and approved the final manuscript.

## Ethics Statement

The Ethics Committee of Peking University approved the CHARLS study, and all participants signed an informed consent form. This study was conducted in accordance with the local legislation and institutional requirements.

## Conflicts of Interest

The authors declare no conflicts of interest.

## Supporting information

Supporting File:

## Data Availability

Data were sourced from the CHARLS database, https://charls.pku.edu.cn/.
