# Supplementary material for: Effect of Small Dense Low‐Density Lipoprotein Cholesterol Combined With High‐Sensitivity C‐Reactive Protein on Cardiometabolic Multimorbidity: A National Cohort Study
Source: Clin Cardiol. 2026 Apr 20;49(4):e70296. doi: 10.1002/clc.70296 (PMC13094409; doi:10.1002/clc.70296)
Supplement: Supplementary file 1 — Supporting File: [file CLC-49-e70296-s001.docx]

**Supplement Table 1**. The difference analysis before and after interpolation of missing values.

| Variables | Before interpolation | After interpolation | Statistics | *P* |
| --- | --- | --- | --- | --- |
| Smoking, n (%) |  |  | χ² = 0.000 | 1.000 |
| No heavy | 7986 (83.05) | 7988 (83.07) |  |  |
| Heavy | 1627 (16.92) | 1628 (16.93) |  |  |
| Gender, n (%) |  |  | χ² = 0.001 | 0.974 |
| Male | 4470 (46.49) | 4470 (46.49) |  |  |
| Female | 5139 (53.44) | 5146 (53.51) |  |  |
| Education, n (%) |  |  | χ² = 0.001 | 0.999 |
| Primary School and Below | 6623 (68.87) | 6630 (68.95) |  |  |
| Junior High School | 1961 (20.39) | 1961 (20.39) |  |  |
| High School and Above | 1025 (10.66) | 1025 (10.66) |  |  |
| Residence, n (%) |  |  | χ² = 0.000 | 1.000 |
| Village | 8665 (90.11) | 8674 (90.20) |  |  |
| Town/City | 942 (9.80) | 942 (9.80) |  |  |
| Insurance, n (%) |  |  | χ² = 0.000 | 1.000 |
| No | 554 (5.76) | 554 (5.76) |  |  |
| Yes | 9053 (94.15) | 9062 (94.24) |  |  |
| eGFR, Mean (±SD) | 96.40 (±13.69) | 96.40 (±13.68) | t = -0.001 | 0.999 |
| Social activity, n (%) |  |  | χ² = 0.000 | 0.993 |
| No | 4585 (47.68) | 4754 (49.44) |  |  |
| Yes | 4686 (48.73) | 4862 (50.56) |  |  |
| Sleeping duration, n (%) |  |  | χ² = 0.200 | 0.905 |
| 6-9 | 4209 (43.77) | 4392 (45.67) |  |  |
| ≤6 | 4615 (47.99) | 4833 (50.26) |  |  |
| >9 | 386 (4.01) | 391 (4.07) |  |  |
| Depression, n (%) |  |  | χ² = 0.103 | 0.749 |
| No | 5537 (57.58) | 6081 (63.24) |  |  |
| Yes | 3252 (33.82) | 3535 (36.76) |  |  |
| Weight, Mean (±SD) | 58.83 (±11.52) | 58.99 (±11.02) | t' = -0.910 | 0.363 |
| Height, M (Q₁, Q₃) | 157.50 (151.80, 164.00) | 157.60 (152.00, 164.10) | W = 39418466.000 | 0.375 |
| Drinking, n (%) |  |  | χ² = 2.499 | 0.114 |
| Less than twice a day | 7781 (80.92) | 9123 (94.87) |  |  |
| More than twice a day | 468 (4.87) | 493 (5.13) |  |  |
| Grip, M (Q₁, Q₃) | 30.00 (23.75, 37.75) | 30.00 (24.00, 37.75) | W = 39080679.500 | 0.341 |

Note: eGFR, estimate glomerular filtration rate.

**Supplement Table 2.** Confounders related to CMM using bidirectional stepwise regression.

| Variables | HR (95% CI) | *P* |
| --- | --- | --- |
| Residence |  |  |
| Village | Ref |  |
| Town/City | 1.15 (0.99-1.32) | 0.059 |
| BMI |  |  |
| <24 | Ref |  |
| ≥24 | 1.32 (1.20-1.45) | <0.001 |
| hsCRP |  |  |
| <3 | Ref |  |
| ≥3 | 1.16 (1.04-1.30) | 0.010 |
| Depression |  |  |
| No | Ref |  |
| Yes | 1.27 (1.15-1.41) | <0.001 |
| Hypertension |  |  |
| No | Ref |  |
| Yes | 1.18 (1.07-1.30) | 0.001 |
| Dyslipidemia |  |  |
| No | Ref |  |
| Yes | 1.30 (1.16-1.45) | <0.001 |
| Arthritis |  |  |
| No | Ref |  |
| Yes | 1.10 (0.98-1.23) | 0.114 |
| Health status |  |  |
| Good | Ref |  |
| Fair | 1.44 (1.23-1.68) | <0.001 |
| Poor | 1.75 (1.48-2.08) | <0.001 |
| Unknown | 1.35 (1.14-1.60) | 0.001 |
| eGFR | 0.91 (0.87-0.95) | <0.001 |
| CMD at baseline |  |  |
| No CMD | Ref |  |
| Diabetes | 6.08 (5.43-6.80) | <0.001 |
| Heart disease | 4.76 (4.19-5.42) | <0.001 |
| Stroke | 2.32 (1.94-2.78) | <0.001 |

Note: CMM, cardiometabolic multimorbidity; BMI, body mass index; hsCRP, high-sensitivity C-reactive protein; eGFR, estimate glomerular filtration rate; CMD, cardiometabolic diseases; HR, hazard ratio; CI, confidence interval; Ref, reference.

**Supplement Table 3.** Testing for multicollinearity among the variables included in the multivariable model.

| Variables | Variance inflation factor (VIF) |
| --- | --- |
| Residence-Town/City | 1.0166 |
| BMI ≥24 | 1.0861 |
| hsCRP ≥3 | 1.0247 |
| Depression-Yes | 1.1575 |
| Hypertension-Yes | 1.081 |
| Dyslipidemia-Yes | 1.082 |
| Arthritis-Yes | 1.0388 |
| Health status-Fair | 2.7636 |
| Health status-Poor | 2.8451 |
| Health status-Unknown | 2.2517 |
| eGFR | 1.046 |
| CMD at baseline-Diabetes | 1.3 |
| CMD at baseline-Heart disease | 1.3279 |
| CMD at baseline-Stroke | 1.1133 |
| Lipid lowering drug-Yes | 1.0836 |

Note: BMI, body mass index; hsCRP, high-sensitivity C-reactive protein; eGFR, estimate glomerular filtration rate; CMD, cardiometabolic diseases;

A VIF-value < 5 indicates the absence of multicollinearity.
